# Diversity and Contributions to Nitrogen Cycling and Carbon Fixation of Soil Salinity Shaped Microbial Communities in Tarim Basin

**DOI:** 10.3389/fmicb.2018.00431

**Published:** 2018-03-09

**Authors:** Min Ren, Zhufeng Zhang, Xuelian Wang, Zhiwei Zhou, Dong Chen, Hui Zeng, Shumiao Zhao, Lingling Chen, Yuanliang Hu, Changyi Zhang, Yunxiang Liang, Qunxin She, Yi Zhang, Nan Peng

**Affiliations:** ^1^State Key Laboratory of Agricultural Microbiology, College of Life Science and Technology, Huazhong Agricultural University, Wuhan, China; ^2^Center for Genome Analysis, ABLife Inc., Wuhan, China; ^3^Agricultural Bioinformatics Key Laboratory of Hubei Province, College of Informatics, Huazhong Agricultural University, Wuhan, China; ^4^Laboratory for Genome Regulation and Human Health, ABLife Inc., Wuhan, China; ^5^Hubei Key Laboratory of Edible Wild Plants Conservation & Utilization, College of Life Sciences, Hubei Normal University, Huangshi, China; ^6^Carl R. Woese Institute for Genomic Biology, University of Illinois at Urbana-Champaign, Urbana, IL, United States; ^7^Department of Biology, Archaeal Centre, University of Copenhagen, Copenhagen, Denmark

**Keywords:** Tarim Basin, microbial community, high-throughput sequencing, halophiles, nitrogen cycle, carbon fixation

## Abstract

Arid and semi-arid regions comprise nearly one-fifth of the earth's terrestrial surface. However, the diversities and functions of their soil microbial communities are not well understood, despite microbial ecological importance in driving biogeochemical cycling. Here, we analyzed the geochemistry and microbial communities of the desert soils from Tarim Basin, northwestern China. Our geochemical data indicated half of these soils are saline. Metagenomic analysis showed that bacterial phylotypes (89.72% on average) dominated the community, with relatively small proportions of Archaea (7.36%) and Eukaryota (2.21%). Proteobacteria, Firmicutes, Actinobacteria, and Euryarchaeota were most abundant based on metagenomic data, whereas genes attributed to Proteobacteria, Actinobacteria, Euryarchaeota, and Thaumarchaeota most actively transcribed. The most abundant phylotypes (*Halobacterium, Halomonas, Burkholderia, Lactococcus, Clavibacter, Cellulomonas, Actinomycetospora, Beutenbergia, Pseudomonas*, and *Marinobacter*) in each soil sample, based on metagenomic data, contributed marginally to the population of all microbial communities, whereas the putative halophiles, which contributed the most abundant transcripts, were in the majority of the active microbial population and is consistent with the soil salinity. Sample correlation analyses according to the detected and active genotypes showed significant differences, indicating high diversity of microbial communities among the Tarim soil samples. Regarding ecological functions based on the metatranscriptomic data, transcription of genes involved in various steps of nitrogen cycling, as well as carbon fixation, were observed in the tested soil samples. Metatranscriptomic data also indicated that Thaumarchaeota are crucial for ammonia oxidation and Proteobacteria play the most important role in other steps of nitrogen cycle. The reductive TCA pathway and dicarboxylate-hydroxybutyrate cycle attributed to Proteobacteria and Crenarchaeota, respectively, were highly represented in carbon fixation. Our study reveals that the microbial communities could provide carbon and nitrogen nutrients for higher plants in the sandy saline soils of Tarim Basin.

## Introduction

One-fifth of the Earth's land surface area is considered desert or dry/arid lands (Laity, 2009), and a greater proportion of these dry/arid lands are under continual threat of desertification as a result of human activities and climate change (Wang et al., 2006; D'Odorico et al., 2013). Distribution of higher plants and animals are limited by extreme environmental conditions in desert or dry/arid lands; therefore, microbial communities are probably the dominant drivers mediating key ecosystem processes in these environments (Makhalanyane et al., 2015). Bacteria, archaea, and fungi are found in desert soils worldwide. Bacterial communities in desert soils typically consist of a number of ubiquitous phyla including Actinobacteria, Bacteroidetes, and Proteobacteria (Chanal et al., 2006; Connon et al., 2007; Lester et al., 2007; Fierer et al., 2009). Based on phylogenetic surveys, among these bacteria, Actinobacteria are predominant in the desert soil (Liu et al., 2009; Makhalanyane et al., 2013; Goswami et al., 2014), probably because of their capacity for sporulation, wide metabolic range, competitive advantages via secondary metabolite synthesis, and multiple UV repair mechanisms (Ensign, 1978; McCarthy and Williams, 1992; Chater and Chandra, 2006; Gao and Garcia-Pichel, 2011). Proteobacteria are also thought to be prominent members of desert soil bacterial communities and might be functionally important in nutrient-limited desert or dry/arid environments, as members of this phylum are implicated in bacteriochlorophyll-dependent photosynthesis (Bryant et al., 2007). Other bacteria identified in desert or dry/arid environments include Cyanobacteria, Firmicutes, and Gemmatimonadetes (Makhalanyane et al., 2015). Cyanobacteria are associated with biogeochemical cycles such as nitrogen or carbon utilization and stress response (Singh et al., 2010, 2013; Chan et al., 2013; Chen et al., 2013). Members of the Firmicutes phylum are also well represented in desert soils (Chanal et al., 2006; Lester et al., 2007; Gommeaux et al., 2010), and a high abundance of Gemmatimonadetes was found to be significantly correlated with low soil moisture (DeBruyn et al., 2011). Fungi including Basidiomycota and Ascomycota, with high taxonomic diversity, are ecologically important for desert systems (Makhalanyane et al., 2015). They can release bioavailable nutrients and associate with desert plants to enhance their colonization and development (Tarafdar et al., 1988; Carrillo-Garcia et al., 1999; Shi et al., 2007). Archaea are commonly found in desert soils with Thaumarchaeota being the principal phylum (Fierer et al., 2012). All known Thaumarchaeota species are chemolithoautotrophic ammonia-oxidizers that might play important roles in biogeochemical nitrogen cycling (Könneke et al., 2005; Brochier-Armanet et al., 2008). Moreover, in some cases, for example, halophilic Euryarchaeota can comprise up to 40% of soil prokaryotic phylotypes in saline soils, as revealed by metagenomic sequencing (Pandit et al., 2014).

Soil microorganisms are important for the stability and productivity of desert ecosystems (Pointing et al., 2007; Makhalanyane et al., 2015). Biocrusts and hypoliths have been shown to stabilize the soil against wind and water erosion, and increase soil fertility and soil moisture, facilitating the growth of plants (Pointing and Belnap, 2012). However, the functions of microbial communities in desert soils are still not well understood. For example, arbuscular mycorrhizal fungi (AMF) are the predominant fungi in desert soil; CO_2_ enhancement of AMF results in considerable soil carbon losses, which challenge the assumption that AMF protect against the degradation of organic carbon in soil (Cheng et al., 2012). Therefore, uncovering the functional roles of microbial communities in desert soil is important for our understanding of chemical cycling in extreme environments.

The majority of previous studies that have focused on the diversity and roles of microbial communities in desert ecosystems are derived from a limited number of desert sites, mainly in USA, Chile-Peru and Australia; however, many other deserts, in particular in Asia and Africa, remain unexplored (Makhalanyane et al., 2015). In this study, we intended to reveal the microbial community structure and its ecological functions in the Tarim Basin, the largest inland Basin in Xinjiang Uyghur Autonomous Region, China, most of which is covered by the world's second largest shifting sand desert, the Taklimakan Desert (Zu et al., 2003; Sun and Liu, 2006). The Tarim Basin has a continental, extreme arid climate featuring hot summers, cold winters, rare precipitation, and strong evaporation. The mean annual temperature varies from 10.6 to 11.5°C with 43.6°C being the maximum and −27.5°C being the minimum; the mean annual precipitation is 116.8 mm and only 50–80 mm in the pediments and 10 mm in the central part of the Basin (Xu et al., 2013). The revolution of high-throughput sequencing techniques during the last decade (Shendure and Ji, 2008), has improved understanding of the community structures and functions of microorganisms in complicated environments. In the present work, we collected microbial communities residing in saline soils of different locations of the Tarim Basin. Genomic DNA and mRNA transcripts of the microbial communities from each location were prepared and subjected to library preparation and high-throughput sequencing. The combinatory analysis of the metagenomic and metatranscriptomic data indicates that the soil salinity shapes microbial communities in Tarim Basin and those communities play important roles in nitrogen metabolism and carbon fixation.

## Materials and methods

### Sample collection and soil chemistry

**Figure 1:**
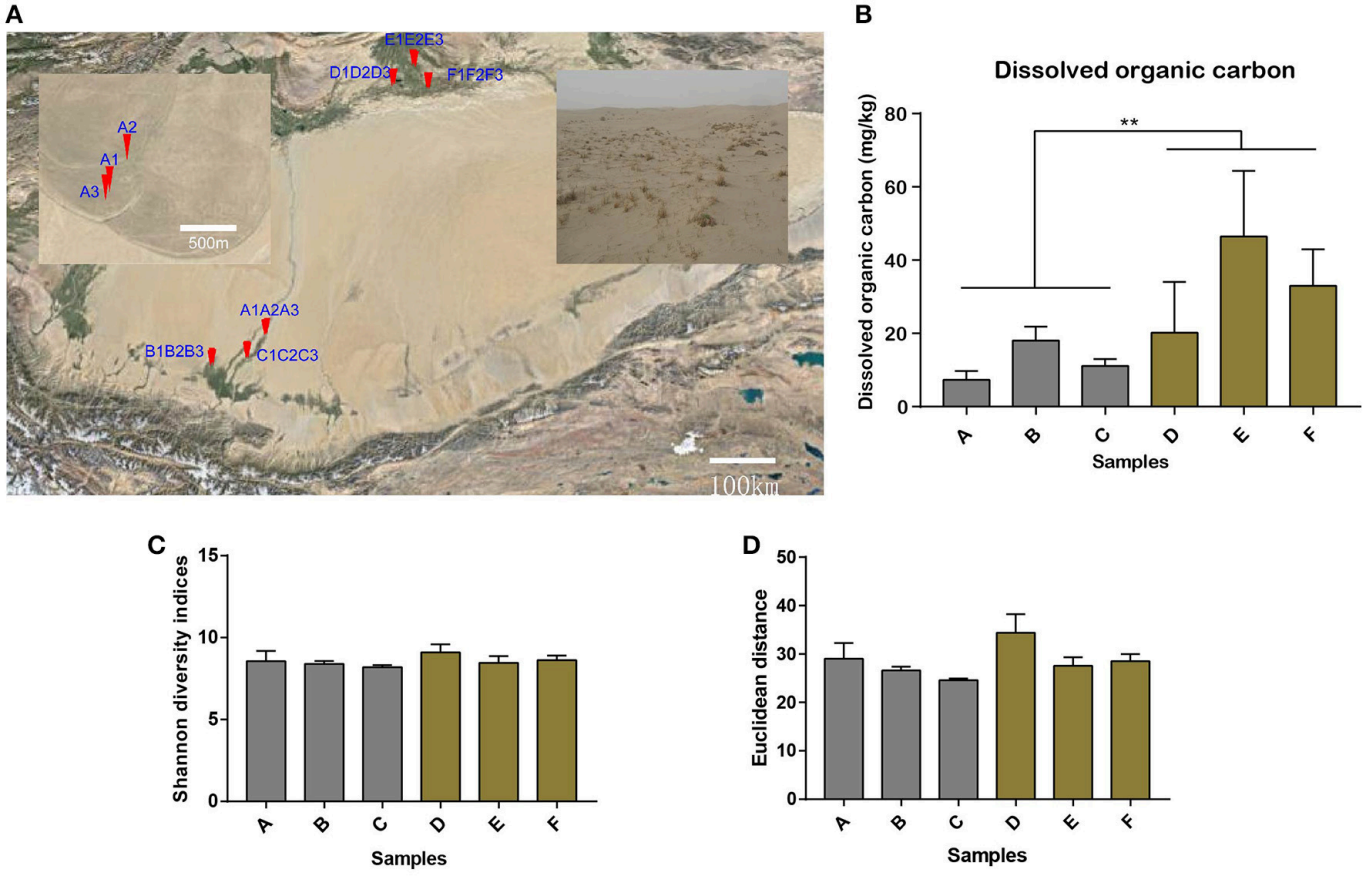
Soil sampling sites in the Tarim Basin. **(A)** The soil samples (*n* = 18) were collected from different oases of the Hotan Prefecture in the south of the Tarim Basin (samples A1–A3, B1–B3, and C1–C3) and from Xayar County in the north of the Tarim Basin (samples D1–D3, E1–E3, and F1–F3). The A sites are shown in the enlarged window. Details of the sample collection sites can be found in Table S1. Soil samples were collected from depths of 10–20, 20–30, and 30–40 cm for each sample. Bar plot showing **(B)** the dissolved organic carbon content (^**^Indicates significant difference) and **(C)** the alpha diversity by Shannon diversity indices for all the 18 samples, as well as **(D)** the consistent beta diversity by Euclidean distance for the three samples in one location for the six sample groups.

Sand soil samples (*n* = 18) were collected from different oases of the Tarim Basin, in April, 2015. Samples A (A_1_, A_2_, and A_3_), B (B_1_, B_2_, and B_3_), and C (C_1_, C_2_, and C_3_) were collected from Hotan Prefecture in the south of the Tarim Basin and samples D (D_1_, D_2_ and D_3_), E (E_1_, E_2_, and E_3_), and F (F_1_, F_2_, and F_3_) from Xayar County in the north of the Tarim Basin (Figure 1). Details of sample collection sites can be found in Table S1. At each sampling site, surface sand was scraped away and a hole with a depth of 50 cm was dug using a sterile steel shovel. Then ca. 30 g soils from each depth at 10–20, 20–30, 30–40 cm were collected and mixed to store in three 50-mL sterile centrifuge tubes. Tubes were covered by dry ice to transfer to the laboratory and stored at −80°C. Then total DNA and total RNA were immediately extracted.

Sand soils were also collected for geochemistry studies. Methods for analysis of conductivity, dissolved organic carbon, total organic carbon, pH, total nitrogen, nitrate nitrogen, ammonium nitrogen, exchangeable potassium, exchangeable sodium, exchangeable calcium, and exchangeable magnesium were described previously (Pansu and Gautheyrou, 2006). The conductivity was measured using a conductivity meter (Leici DDS307, INESA instrument) after aqueous extraction. The dissolved organic carbon was measured by a TOC analyzer (vario PYRO cube, Elementar,), and the total organic carbon was measured by the potassium dichromate heating method. The soil pH was measured using a pH meter (Leici PHS-3C, INESA instrument) after shaking the sample for 1 min with ddH_2_O and allowing it to stand for 30 min. Total soil nitrogen was measured by the Semi-micro Kjeldahl method after sulfuric acid leaching. Nitrate nitrogen and ammonium nitrogen were measured by a colorimeter (UV759S, Shanghai TecFront) after KCl extraction. Exchangeable potassium and sodium were measured by a Model 410 flame photometer (Sherwood scientific LTD) after ammonium acetate extraction. Exchangeable calcium and magnesium were measured by an atomic absorption spectrophotometer (G8432A, Agilent Technologies) after ammonium acetate extraction.

### Extraction of nucleic acids, library construction, and sequencing

Equal mass (5 g) of soil samples from three different depths at each sampling site were mixed and were to be used for nucleic acid extraction. Genomic DNA was extracted from soil samples using the E.Z.N.A.™ Soil DNA Kit (Omega), following the manufacturer's instructions. The quality and quantity of genomic DNA were determined by measuring the absorbance at 260/280 nm (A_260_/A_280_) using the SmartSpec Plus Spectrophotometer (Bio-Rad). DNA integrity was further verified by 1.0% agarose gel electrophoresis. For each sample, 100 ng of fragmented genomic DNA was used for library preparation. DNA was fragmented to 100–400 bp by sonication with an M220 Focused-ultrasonicator (Covaris). DNA end repairing and A-tailing was performed with a Kapa HTP Library Prep Kit for Illumina platform (Kapa). The A-tailed DNA was ligated with adaptors and a library containing ideal fragment sizes (300–500 bp) was obtained using the SeqCap Adapter Kit A (Kapa).

Total RNA was extracted from soil samples using the E.Z.N.A.™ Soil RNA Kit (Omega), following the manufacturer's instructions, and was treated with RQ1 DNase (Promega) to remove trace DNA. The quality and quantity of the purified RNA were determined by measuring the absorbance at 260/280 nm (A_260_/A_280_) using the SmartSpec Plus Spectrophotometer (Bio-Rad). RNA integrity was further verified by 1.5% agarose gel electrophoresis. For each sample, 120 ng of total RNA was used for library preparation. Ribosomal RNA was depleted using the Ribo-Zero Magnetic Gold Kit (Epicentre). Purified RNA was fragmented at 85°C. Reverse transcription was performed with random primers harboring the adapter sequence and a randomized hexamer provided by the ScriptSeq™ V2 RNA-Seq Library Preparation Kit (Epicentre). cDNA was amplified with a ScriptSeq Index PCR primers kit (Epicentre). Size selection (300–500 bp) of the cDNA library was performed using Gnome DNA Clean (Gnomegen), a magnetic bead-based DNA purification system. For high-throughput sequencing, the libraries were subjected 150-nt paired-end sequencing using the Illumina NextSeq 500 system by ABlife Inc. (Wuhan, China).

### Assembly and bioinformatics analyses

#### Raw read filtering

Raw reads were first discarded if they contained more than 2-N bases; filtered reads were obtained by trimming adaptor sequences, removing low quality bases, and discarding reads of less than 16-nt using the FASTX-Toolkit (Version 0.0.13).

#### Contig assembly

Clean reads were assembled using the *de novo* assembler Meta-Velvet (Namiki et al., 2012) at a k-mer of 30. The 200-bp length threshold was set to filter the short contigs after assembly. Contigs generated by each assembly were combined and cd-hit software (Li and Godzik, 2006) was utilized to obtain final, non-redundant contigs. After assembly, open reading frames (ORFs) were predicted using MGA (Noguchi et al., 2008), and a BLAST search (Altschul et al., 1990) was conducted on the ORF sequences, comparing to NCBI's non-redundant protein (“nr”) and Kyoto Encyclopedia of Genes and Genomes (KEGG) databases (Kanehisa and Goto, 2000) with an E-value cut-off of 1e10^−5^ to obtain functional annotation.

Filtered cDNA reads were mapped to the final ORF sequence file using Bowtie (Langmead et al., 2009), allowing two mismatches. The abundance of each ORF was obtained from the mapping results by calculating RPKM values (Mortazavi et al., 2008). Based on the taxonomic information from the NCBI Taxonomy database (http://www.ncbi.nlm.nih.gov/taxonomy), the abundance of each operational taxonomic unit (OTU) was obtained according to the summation of genes originating from the same species.

### MetaphlAn2 and graphlan analysis

For both metagenomic and metatranscriptomic reads, MetaPhlAn2 and GraPhlAn software (Truong et al., 2015) were utilized to obtain the relative abundance of each OTU. The top 50 (relative abundance) OTUs were chosen and displayed in a dendrogram heatmap. Circular taxonomy clustering tree was generated by GraPhlAn based on species with an abundance between 30 and 300.

### *De novo* OTU picking and statistical analysis

The filtered reads from metagenomics samples were aligned to the Silva database (Quast et al., 2013) to calculate the rDNA abundance. We extracted the V3-V5 region of 16S rDNAs which had no less than two aligned reads. The selected rDNA sequences were then classified into OTUs at a threshold of 97% sequence identity using UCLUST embedded in QIIME (Caporaso et al., 2010). Alpha and beta diversities were performed by Shannon diversity indices and Euclidean distance, respectively.

### Other statistical methods

To test for statistical differences of each chemistry characteristic between Xayar and Hotan, we performed a two-way analysis of variance (ANOVA) method with location (Xayar vs. Hotan) and within location sites (*n* = 3) as treatments for each parameter. Similar tests were made comparing alpha diversity (Shannon) and beta diversity (Euclidean Distance) was also performed by two-way ANOVA. We tested for normality and equal variances of our data before performing the ANOVA. The KEGG database was utilized to define the enrichment of identified genes in each KEGG pathway. Statistical figures and tables were obtained using the R package. Clustering was performed by Cluster3.0 software and heatmaps were generated using Java TreeView (http://bonsai.hgc.jp/~mdehoon/software/cluster/software.htm).

### Data deposition

The sequences reported in this paper have been deposited in the European Nucleotide Archive (accession no. PRJEB21269).

## Results

### Geochemistry of the desert soils in tarim basin

The Tarim Basin, and the Taklamakan desert in its center, is surrounded by high mountains, namely the Tianshan in the north and Kunlun and Altyn ranges in the south. Precipitation in the mountains provides valuable water resources for the plain and piedmont areas, establishing the oases in the river deltas and alluvial–diluvial plains (Hong et al., 2003). In this study, we attempted to reveal the microbial community structures and their ecological functions in nitrogen metabolism and carbon fixation of the soils collected at the edge of the oases in Tarim Basin. Samples were collected from six sites, specifically, three locations in Hotan and another three locations in Xayar (Figure 1A, Table S1). At the sampling sites, plant growth was sparse on the sandy soils (Figure 1A). Geochemistry data were generated for 18 soil samples, for which each sample was mixed and contained soils from three layers of 10–20, 20–30, and 30–40 cm beneath the surface. The total and dissolved organic carbon, total nitrogen, nitrate nitrogen, ammonium nitrogen, exchangeable potassium, exchangeable sodium, exchangeable calcium, exchangeable magnesium, and pH values of the samples are shown in Table 1. We tested the statistical difference for each characteristic by ANOVA method (Table S2). Significant difference was observed between locations for dissolved organic carbon (*p*-value = 0.0025), total nitrogen (*p*-value = 0.0175), nitrate nitrogen (*p*-value = 0.0063), and exchangeable calcium (*p*-value = 0.0008). The nitrate nitrogen also showed difference (*p*-value = 0.0226) between sites. The dissolved organic carbon varied from 5.05 to 66.85 mg kg^−1^, and its concentration in the northern part (Xayar area) of Tarim Basin was higher than that in the southern part (Hotan area) (*p*-value = 0.0025, ANOVA, Figure 1B, and Table 1). Total nitrogen varied from 57.1 to 495.9 mg kg^−1^, which is similar to that of other deserts (Makhalanyane et al., 2015), and also showed significant difference between two locations (Table S2). Besides, the interaction factor of total carbon showed significant difference (*p*-value = 0.0492, Table S2), indicating that the site variation patterns varied between locations. The conductivity of the soils varied from 0.42 to 8.68 dS m^−1^ (Table 1), indicating that two soils (A3 and C3) were of low salinity (conductivity: 2-4 dS m^−1^), whereas seven soils (A1, A2, D3, E1, E2, E3, and F2) were of moderate salinity (Table 1).

**Table 1:** The chemistry characteristics of the 18 oases soil samples.

**Sample**	**Conductivity (dS m^−1^)**	**Dissolved organic carbon (mg kg^−1^)**	**Total carbon (g kg^−1^)**	**pH**	**Total nitrogen (mg kg^−1^)**	**Nitrate nitrogen (mg kg^−1^)**	**Ammonium nitrogen (mg kg^−1^)**	**Exchangeable potassium (mg kg^−1^)**	**Exchangeable sodium (mg kg^−1^)**	**Exchangeable calcium (mg kg^−1^)**	**Exchangeable magnesium (mg kg^−1^)**
A1	7.01	6.97	4.62	8.36	93.00	16.27	8.41	44.62	N.D.	14.04	2.052
A2	4.84	9.86	3.49	8.25	139.30	37.57	18.05	32.23	281.70	14.29	1.487
A3	3.70	5.05	1.78	8.04	109.50	36.00	27.52	44.62	477.70	16.28	1.323
B1	N.D.	N.D.	3.17	N.D.	175.50	N.D.	N.D.	19.83	162.20	N.D.	N.D.
B2	1.28	20.74	1.72	9.18	168.00	37.48	1.40	29.75	157.30	14.30	1.823
B3	0.88	15.31	2.05	8.67	187.40	35.80	9.30	22.31	52.74	14.27	1.838
C1	0.59	8.86	4.42	8.73	66.10	22.39	10.62	9.92	32.83	7.62	0.715
C2	0.42	11.99	2.64	8.74	67.60	26.04	9.15	4.96	16.98	9.58	1.053
C3	2.06	12.36	2.77	8.65	69.10	30.37	9.73	19.83	226.95	9.72	0.885
D1	1.65	29.95	3.50	8.57	189.00	46.94	5.93	17.35	182.10	15.41	1.131
D2	N.D.	N.D.	5.28	N.D.	272.80	N.D.	N.D.	37.71	441.00	N.D.	N.D.
D3	5.67	10.35	2.97	8.14	238.00	38.66	20.28	47.10	427.90	17.93	1.979
E1	8.68	66.85	N.D.	7.99	57.10	28.30	15.46	47.10	N.D.	20.22	3.940
E2	4.55	33.24	5.74	8.03	178.40	57.20	23.71	22.31	253.90	19.51	2.063
E3	4.05	39.29	5.93	7.86	250.50	62.23	20.12	22.31	346.40	19.19	2.012
F1	N.D.	N.D.	6.73	8.75	495.90	22.19	7.00	29.75	95.56	20.08	1.483
F2	4.53	40.05	6.80	8.66	488.50	52.27	37.18	59.50	450.90	19.71	2.533
F3	1.10	25.92	6.26	9.15	311.70	40.83	10.15	14.87	144.80	18.36	1.312

*N.D. not determined*.

### Diversity analysis of sequencing data

To give a more comprehensive understanding of the structure of the microorganisms, we used the next generation sequencing method to capture the structure and abundance of the microorganisms. Metagenomic and metatranscriptomic sequencing generated about 39–46 million and 13–37 million 150-nt reads, respectively, for each sample (Tables S3, S4). After quality filtering, about 85 and 87% reads passed the quality threshold and participated in the following analysis for metagenomic and metatranscriptomic samples, respectively. So we systematically researched the microbial structure by performing the alpha and beta diversity analysis for all the metagenomics samples. By utilizing operational taxonomic units (OTUs) analysis tools embedded in QIIME (Caporaso et al., 2010), we obtained 6,375 OTUs for all the metagenomics samples, implying the high species richness of soil microorganisms. Alpha diversity by Shannon diversity indices, reached over 8 in most samples (Figure 1C), and showed no significant difference between locations (*p*-value = 0.134, ANOVA, Table S2) or sites (*p*-value = 0.659, ANOVA), as well as their interactions (*p*-value = 0.826, ANOVA), indicating that all the soil samples contain abundant microorganisms. Beta diversity by Euclidean distance, representing the diversity between samples, also showed no significant difference (*p*-value = 0.0529, ANOVA, Figure 1D and Table S2) between locations. In summary, the diversity analysis can conclude that microbial community composition in different locations was not significant difference.

### Phylogenetic analysis

**Figure 2:**
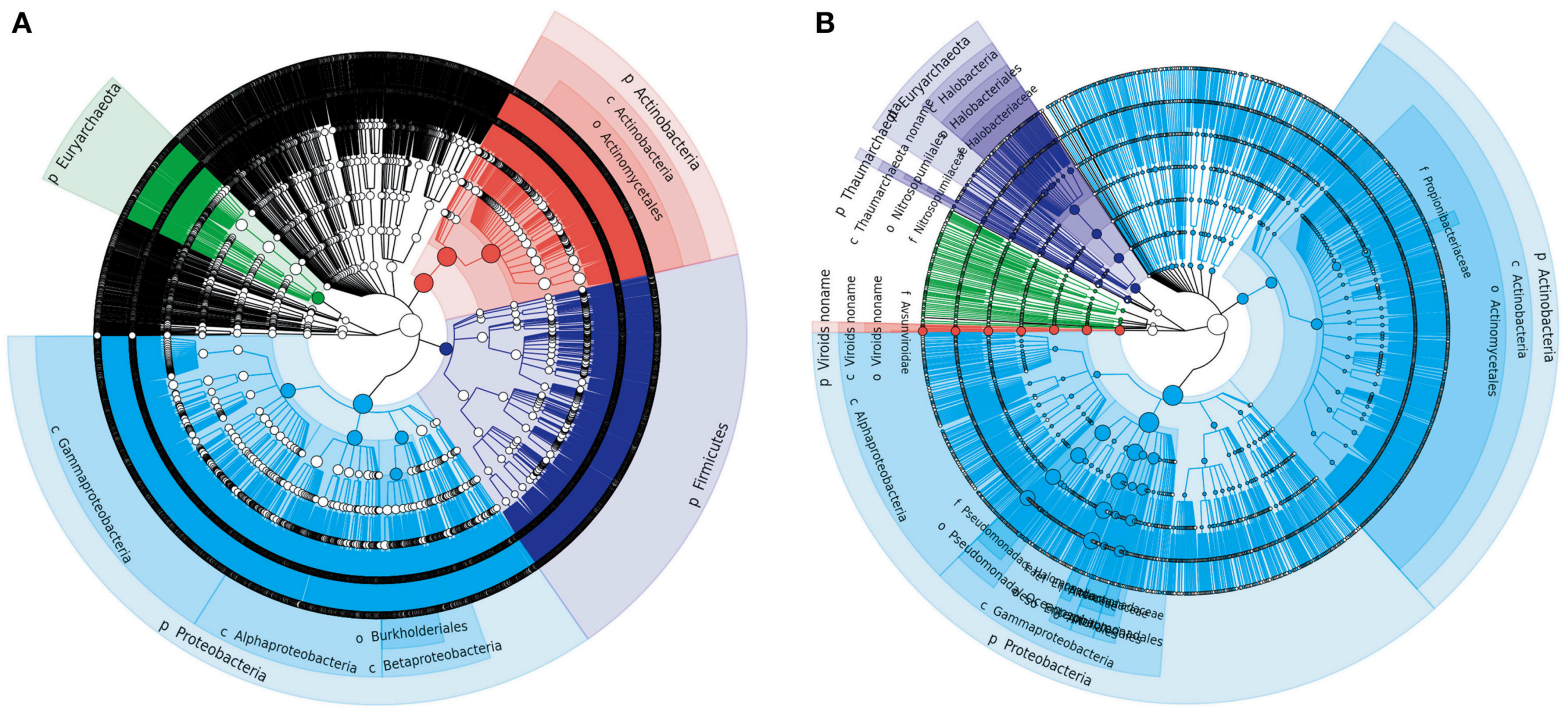
Phylogenetic trees of Tarim microbial species-level OTUs determined by metagenomic **(A)** and metatranscriptomic **(B)** analysis. Soil samples were collected from the Tarim Basin, from different locations and depths, and the associated genomic DNA and transcriptomes were subjected to deep sequencing analysis. Based on the results, a circular taxonomy clustering tree was produced by GraPhlAn.

**Figure 3:**
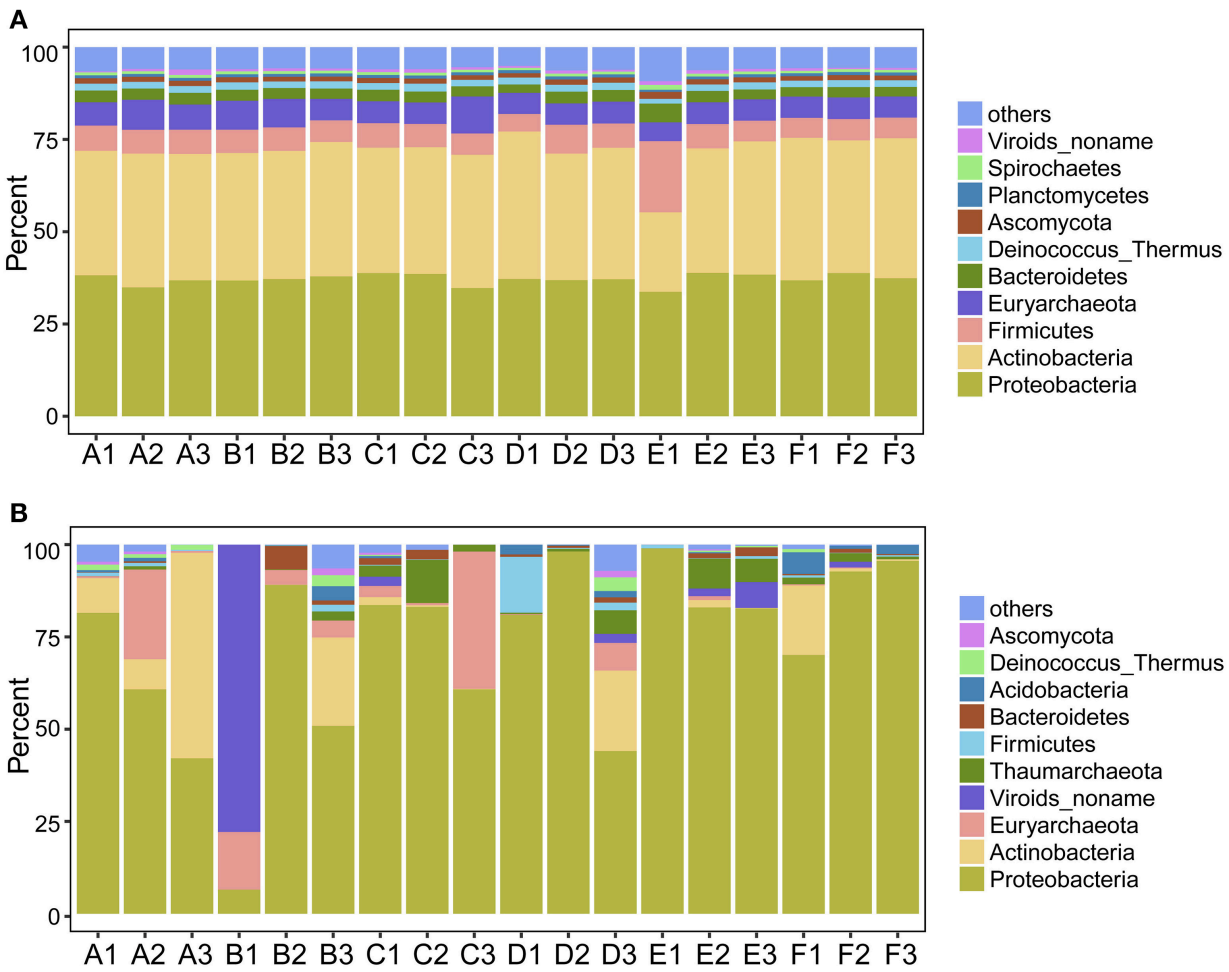
Proportion of the most abundant phylum-level OTUs determined by metagenomic **(A)** and metatranscriptomic **(B)** analysis. Soil samples were collected from the Tarim Basin, from different locations and depths, and associated genomic DNA and transcriptomes were subjected to analysis based on deep sequencing and phylogenetic analysis.

**Figure 4:**
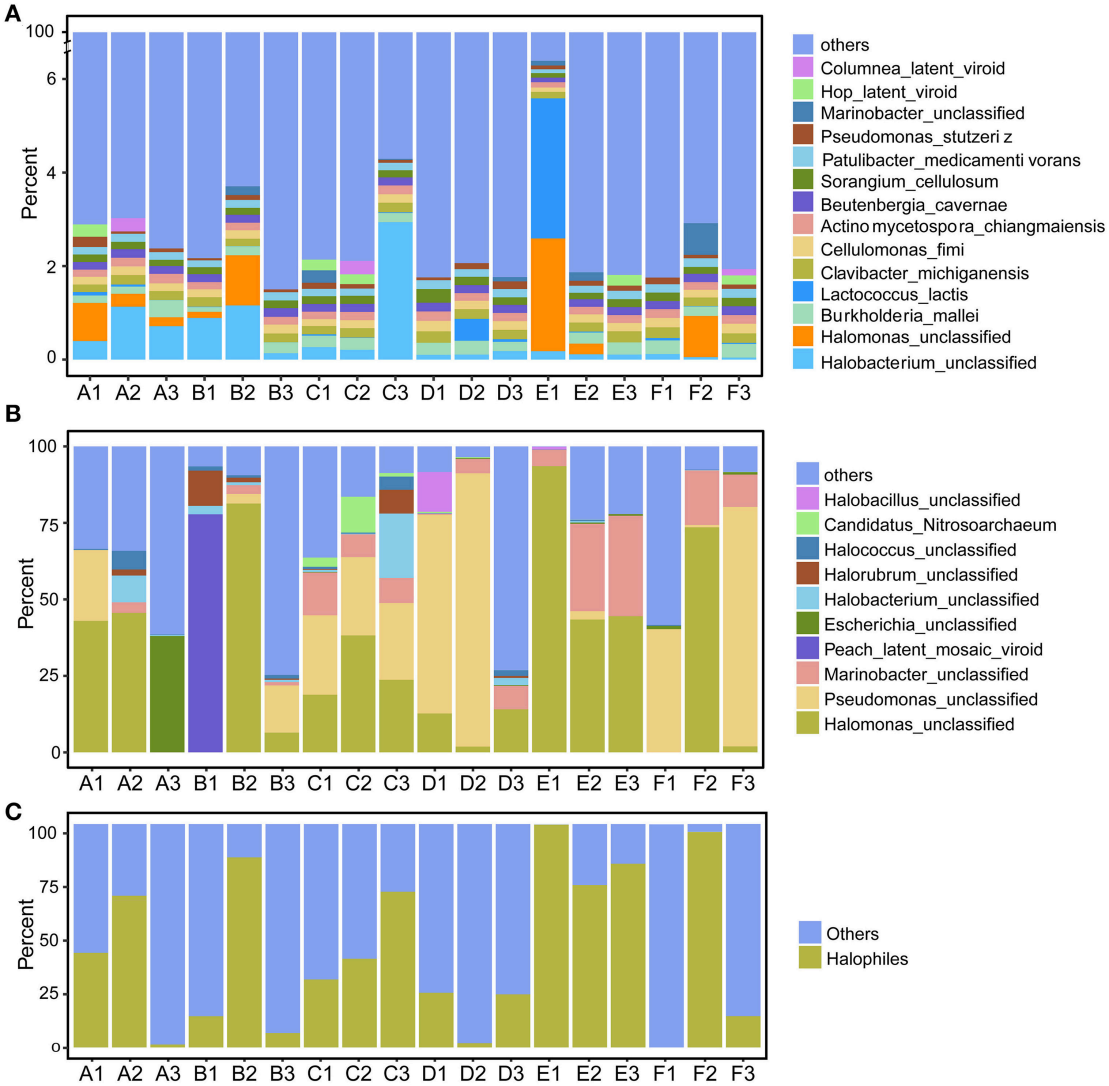
Proportion of the most abundant species-level OTUs, as determined by metagenomic **(A)** and most actively transcribed species by metatranscriptomic **(B)** analysis, and the proportion of putative halophilic microorganisms determined by metatranscriptomics **(C)**. Soil samples were collected from the Tarim Basin, from different locations and depths; genomic DNA and transcriptomes were subjected to deep sequencing and phylogenetic analysis.

**Figure 5:**
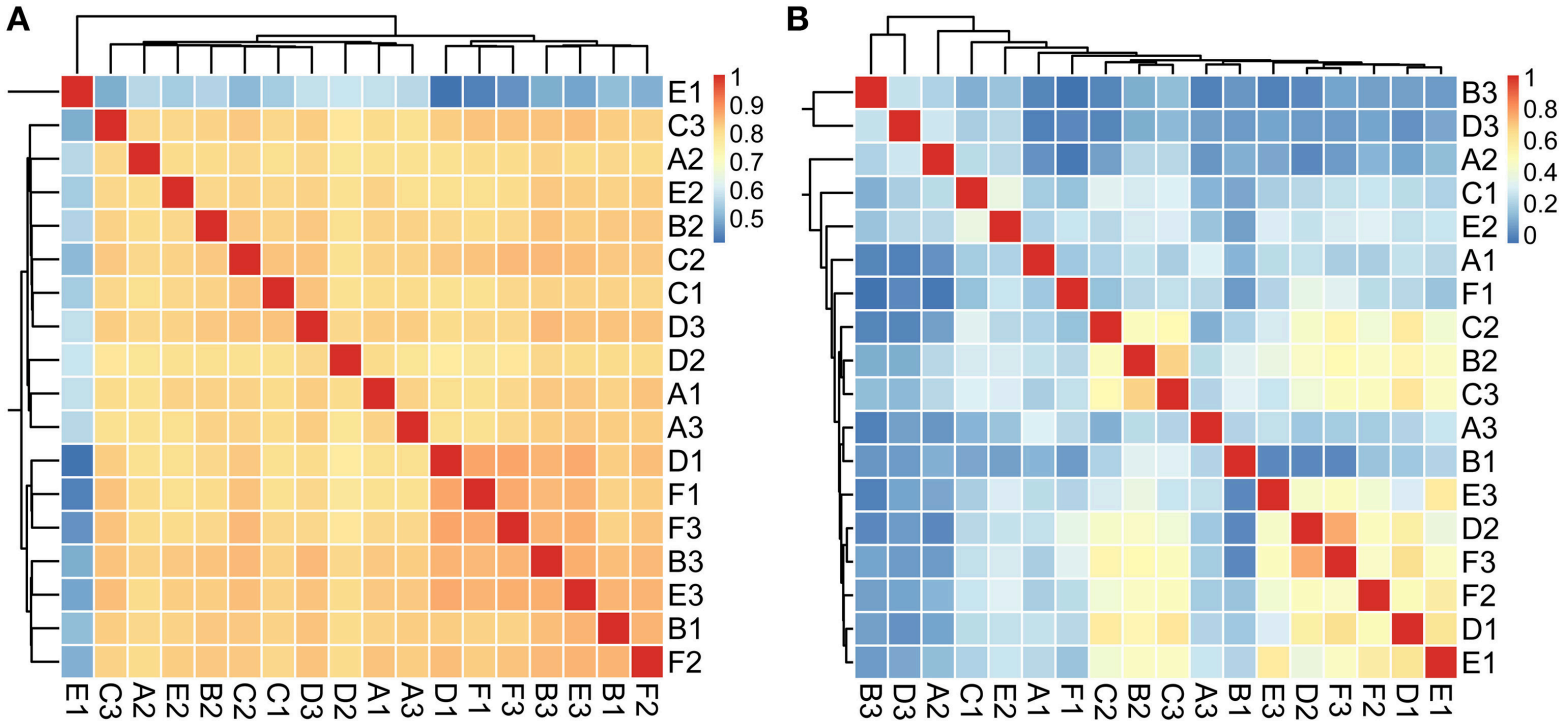
Analysis of the sample correlation according to the detected species in metagenomic **(A)** and metatranscriptomic **(B)** data among the tested soil samples. The correlation value is indicated as the color of light blue (un-related) to pink (related).

We first explored the microbial community compositions by analyzing the metagenomic reads using unique clade-specific marker genes by MetaPhlAn2 (Truong et al., 2015). MetaPhlAn2 analysis of the taxonomic prediction, using metagenomic reads, suggested that the desert soil ecosystems contain very high bacterial abundance (89.72% of the total phylotypic signals on average) with a low proportion of Archaea (7.36%), Eukaryota (2.21%), and Viroids (0.71%) (Figure 2A; Table S5). From the Bacteria domain, Proteobacteria, Actinobacteria, and Firmicutes were predominant in the tested soils, whereas Euryarchaeota of the Archaea domain, mainly comprising *Halorubrum, Haloferax, Halobacterium, Haloarcula*, and *Haloterrigena* species-level OTUs, were the most abundant archaea present (Figure 3A; Table S6). The top 10 species in each sample according to the MetaPhlAn2 analysis of metagenomic reads are shown in Figure 4A and Table S7. *Halomonas, Halobacterium*, and viroids were highly represented in all A samples; *Halomonas, Halobacterium, Halorubrum*, viroids, and *Streptomyces* species were highly represented in all B samples; and *Halobacterium*, viroids, *Burkholderia mallei, Cellvibrio, Cellulomonas, Sorangium cellulosum* were highly represented in all C samples. Furthermore, *Cellvibrio, Cellulomonas, S. cellulosum, Clavibacter michiganensis, B. mallei*, and viroids were highly represented in D samples; *C. michiganensis, B. mallei*, and viroids were highly represented in E samples; and *B. mallei, C. michiganensis*, viroids, *Cellvibrio, Cellulomonas*, and *S. cellulosum* were highly represented in F samples. Overall, *Halobacterium* and viroids were highly represented in A, B, and C samples, which were collected from Hotan, and *C. michiganensis, B. mallei*, and viroids were highly represented in D, E, and F samples, which were collected from Xayar, according to MetaPhlAn2 analysis of metagenomic data. Analysis of the sample correlation according to the detected species-level OTUs in metagenomic data showed significant differences (*p*-value < 0.01), indicating low correlation of species among the soil samples (Figure 5A).

Second, we explored the community composition of transcriptionally active microorganisms by analyzing the metatranscriptomic data using MetaPhlAn2. This showed that the transcripts from Archaea, Bacteria, Eukaryota, and Viroid varied largely in different samples (Figure 2B). Bacteria were most active in all samples; Eukaryota and Viroids were least active in these samples, with the exception of viroids in B1 sample (77.83%), the Archaea domain was less active in A1, A3, D1, D2, E1, and F3 samples (0.05%~0.74%) and active in A2, B1, C2, C3, and D3 samples (12.29%~39.07%) (Table S8). MetaPhlAn2 analysis revealed that genes associated with the Proteobacteria phylum was most actively transcribed in all 18 soil samples, except A3 and B1, whereas genes associated with both Proteobacteria and Actinobacteria were active in the A3 sample (Figure 3B, Table S9). Genes of Actinobacteria was the second-most actively transcribed in A1, B3, D3, and F1 samples. In contrast, genes of viroids were most actively transcribed in the B1 sample, and genes of Euryarchaeota was the second-most actively transcribed in A2, B1, and C3 samples (Figure 3B, Table S9). Genes of other phyla such as Firmicutes, Euryarchaeota, Bacteroidetes, Deinococcus–Thermus, and Ascomycota were less transcribed in the total microbial population (Figure 3B, Table S9). Upon comparing phyla based on the MetaPhlAn2 analysis results from the metatranscriptomic reads, genes associated with Proteobacteria were determined to be most actively transcribed, whereas genes of Actinobacteria were less transcribed in almost all samples.

### Halophiles are the most active species in tarim soils

The 10 most active species-level OTUs from each sample, based on DNA content analysis of species by transcripts per million (TPM, TPM [species] = reads [species] × 1000,000/total mapped reads) are shown in Figure 4B and Table S10. Surprisingly, the 10 most active species represented a large proportion of total active species in all tested soil samples (Figure 4B). A *Halomonas* species was most active in A1 and A2 samples, and an unclassified *Escherichia* species was most active the A3 sample (Figure 4B; Table S10). A *Halomonas* species was most active in the B2 sample and *Pseudomonas* and *Halomonas* species were actively transcribed in B3 and all C samples (Figure 4B; Table S10). *Pseudomonas, Halobacillus*, and *Halomonas* species were active in D1; *Pseudomonas* species, and *Halomonas* and *Marinobacter* species were most active in D2 and D3, respectively (Figure 4B; Table S10). *Halomonas* and *Marinobacter* species were active in all E samples, and *Pseudomonas* species were active in F1 and F3 samples, whereas *Halomonas* species were active in F2 sample. In summary, based on the similarity to known halophilic genera, we found halophiles were most active in most soil samples, especially in A1-2, B2, C1-3, E1-3, and F2 samples (Figure 4C). Moreover, analysis of the sample correlation according to the active species showed significant differences (*p*-value < 0.01), indicating high diversity of active species among the soil samples (Figure 5B).

We further analyzed the transcripts of the most active halophilic species, closely related to *Halobacterium* sp. DL1 and *Halomonas elongate* DSM 2581, to understand their adaptation to the arid environment of Tarim Basin. Importantly, stress responsive proteins, e.g., cold-shock proteins, stress response translation initiation inhibitor, thermosome subunits (the archaeal molecular chaperones), cation transporters, were found to be highly expressed in the *Halobacterium* species (Table S11). Similarly, the stress responsive proteins (cold-shock proteins and molecular chaperone), as well as gene expression proteins (RNA polymerase subunits and ribosome proteins) were highly expressed in the *H. elongate* species (Table S11). All these results indicate that these halophilic archaeal and bacterial species are adapted to the thermal and arid conditions by high expression of stress responsive proteins.

### Analysis of active nitrogen metabolism pathways

**Figure 6:**
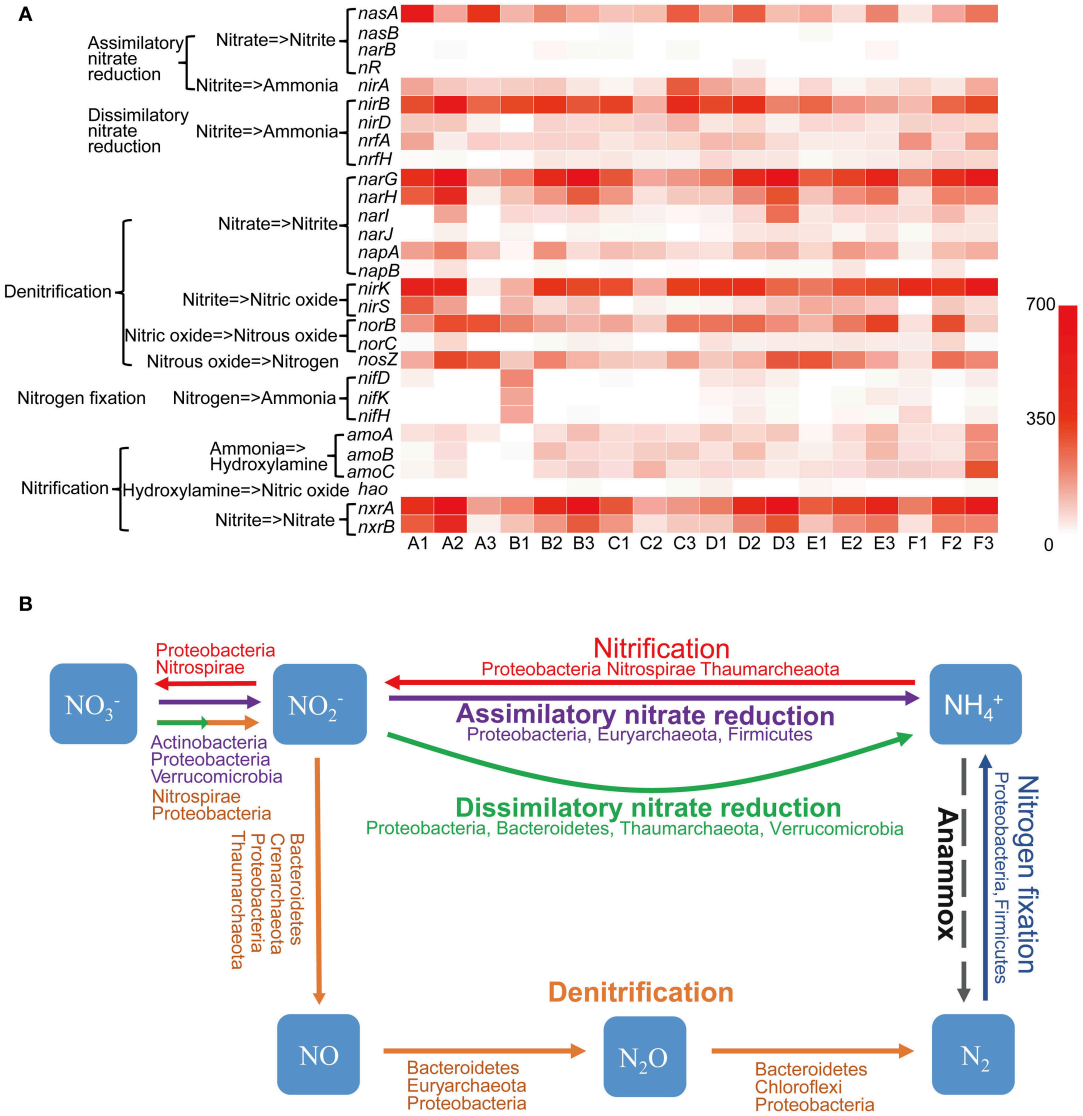
Nitrogen cycle in Tarim saline soils. **(A)** Heat map showing the metatranscriptomic reads of genes involved in the nitrogen cycle based on deep sequencing data obtained from the soil samples. *nasA* and *B*, assimilatory nitrate reductase subunits; *narB*, ferredoxin-nitrate reductase; *nR*, nitrate reductase; *nirA, B* and *D*, nitrite reductase subunits; *nrfA* and *H*, cytochrome c nitrite reductase subunits; *narG, H, I*, and *J*, nitrate reductase subunits; *napA* and *B*, periplasmic nitrate reductase subunits; *nirK* and *S*, nitrite reductase subunits; *norB* and *C*, nitric oxide reductase subunits; *nosZ*, nitrous-oxide reductase; *nifD, K* and *H*, nitrogenase molybdenum-iron protein subunits; *amoA, B* and *C*, ammonia monooxygenase subunits; *hao*, hydroxylamine dehydrogenase; *nxrA* and *B*, nitrite oxidoreductase subunits. **(B)** Schematic representation of the nitrogen cycling. Phyla importantly contributed to the pathways are indicated. Dash arrow indicates the Anammox pathway is not detected in Tarim soil samples.

Aside from water, nitrogen is often regarded as the most limiting factor of productivity in arid terrestrial ecosystems (Hooper and Johnson, 1999). Nitrogen metabolism genes were found to be actively expressed in Tarim soils, according to the transcriptome data. Genes involved in five nitrogen metabolism pathways including assimilatory nitrate reduction, dissimilatory nitrate reduction, denitrification, nitrogen fixation, and nitrification pathways, were detected (Figure 6A). The assimilatory nitrate reduction pathway includes the *nasA, nasB, narB, nR*, and *nirA* genes. The *nasA* gene product catalyzes the first step, specifically the conversion of nitrate (NO^3−^) to nitrite (NO^2−^), and the *nirA* gene product catalyzes the nitrite to ammonium reaction; in terms of the assimilatory nitrate reduction pathway, these genes were well-represented in all 18 soil samples. However, *nasB, narB*, and *nR* were not as well represented in this pathway (Figure 6A). Proteobacteria contributed mostly to *nasA* gene expression (Figure 6B, Table S12). Strains that contributed to *nasA* gene transcription varied in all 18 Tarim soil samples, even in closely located samples (Table S12). The *nasB* gene was dramatically underrepresented (Figure 6A) and was only contributed by an unknown strain and *Streptomyces rimosus* (Table S12). *Halobacteria* of Euryarchaeota contributed mostly to *nirA* gene expression in A (63.74% for A1, 86.97% for A2), B (32.99% for B1, 47.08% for B2), C (65.45% for C1, 18.58% for C2, and 74.65% for C3), and E1 (26.32%) samples (Figure 6B, Table S12). Firmicutes and Verrucomicrobia also contributed a large proportion to *nirA* expression (Figure 6B, Table S12).

The dissimilatory nitrate reduction pathway includes the *nirB, nirD, nrfA*, and *nrfH* genes. These genes were actively transcribed in all 18 samples; however, *nrfH* was less transcribed in A1, A2, A3, B1, C3, and E1 samples (Figure 6A). Proteobacteria and Bacteroidetes contributed mostly to *nirB, nirD*, and *nrfA* gene expression (Figure 6B, Table S12). However, Thaumarchaeota and Verrucomicrobia also contributed a large proportion to *nirD* and *nrfA* expression, respectively (Table S12). The *nrfH* gene was less expressed in soil samples and Proteobacteria and Planctomycetes contributed mostly to its expression (Table S12).

Denitrification, the biological production of NO, N_2_O, and N_2_ gases from ${\text{NO}}_{3}^{-}$ under anoxic condition, is a key process that contributes to the nitrogen cycle. This pathway includes the *narGHIJ, napAB, nirKS, norBC*, and *nosZ* genes (Figure 6A). In the denitrification pathway, all genes were actively transcribed, except *narJ, napB*, and *norC*, which are involved in the catalysis of nitrate to nitrite, and in the catalysis of nitrous oxide (N_2_O) to nitrogen in some soil samples (Figure 6A). Proteobacteria were the most important contributor of all genes involved in this pathway, whereas Nitrospirae, Bacteroidetes, and unknown or uncultured microorganisms also were shown to play important roles in denitrification (Table S12). Importantly, Thaumarchaeota contributed significantly to the presence of *nirK*, whereas Crenarchaeota and Euryarchaeota contributed significantly to the presence of *nirK* and *norB* in some samples such as A1, A2, B2, D1, and F3 (Table S12).

The nitrogen fixation pathway was not active in A, B, and C soil samples, with the exception of B1, but was present in D, E, and F soil samples (Figure 6A). Proteobacteria and Firmicutes were the most important contributors to this pathway (Table S12).

The nitrification pathway includes ammonia oxidation and nitrite oxidation. Ammonia oxidation comprises two steps: the first step of this process is catalyzed by ammonia monooxygenase (Amo), producing hydroxylamine (NH_2_OH), and hydroxylamine oxidoreductase (Hao) then further oxidizes NH_2_OH to NO (Caranto and Lancaster, 2017). The *amoA, amoB*, and *amoC* were mainly expressed by Thaumarchaeota in almost all soil samples except A3 and B1, whereas *hao* mainly expressed by ammonium-oxidizing bacteria was only marginally transcribed in all 18 soil samples (Figure 6A). These results indicated that Archaea played the most important role in ammonia oxidation in Tarim saline soil samples. Species of Thaumarchaeota and an uncultured archaeon contributed to most *amoA* and *amoB* genes expression, whereas Thaumarchaeota contributed a large proportion to *amoC* expression (Table S12). The *hao* gene was transcribed at a very low level (Figure 6A), and was contributed by Proteobacteria and Nitrospirae. In summary, Thaumarchaeota was the most important contributor to ammonia oxidation in Tarim soils. The *nxrA* and *nxrB* genes, which encode products that catalyze the conversion of nitrite to nitrate in the nitrification pathway were highly active in almost all soil samples (Figure 6A). Proteobacteria were the most important contributor of *nxrA* and *nxrB*, whereas Nitrospirae also contributed a large proportion to *nxrA* and *nxrB* expression (Table S12). However, the anaerobic ammonium oxidation (Anammox) pathway, which seems to be of ecological importance in marine environments (Oshiki et al., 2016), was not detected in any of the 18 soil samples. These results indicated that dissimilatory nitrate reduction, denitrification, and nitrification pathways were active in microbial communities in the saline soils of the Tarim Basin, and played important roles in the nitrogen cycles.

### Carbon fixation by microbial communities of the tarim basin soils

**Figure 7:**
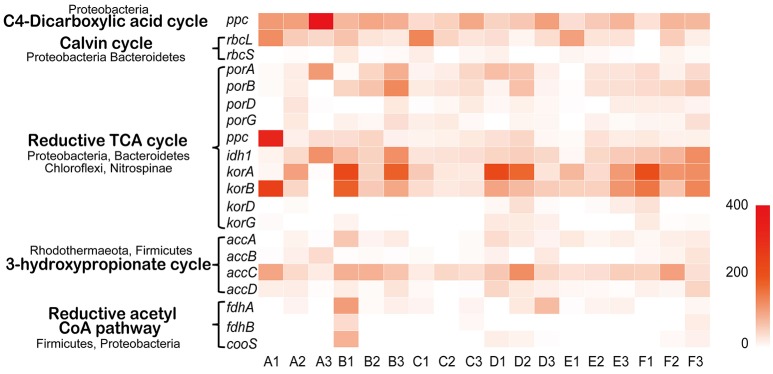
Carbon fixation in Tarim saline soils. Heat map showing the metatranscriptomic reads of genes for carbon fixation based on deep sequencing data obtained from soil samples. The contributing phyla are indicated. *Ppc*, phosphoenolpyruvate carboxylase; *rbcL* and *S*, ribulose-bisphosphate carboxylase subunits; *porA, B, D* and *G*, pyruvate-ferredoxin oxidoreductase subunits; *idh1*, isocitrate dehydrogenase; *korA, B, D* and *G*, 2-oxoglutarate/2-oxoacid ferredoxin oxidoreductase subunits; *accA, B, C* and *D*, acetyl-CoA carboxylase subunits; *fdhA* and *B*, formate dehydrogenase subunits; *cooS*, anaerobic carbon-monoxide dehydrogenase catalytic subunit.

The transcripts of key genes encoding components of carbon fixation pathways were detected in Tarim saline soils. The carbon fixation pathways include the C4 dicarboxylic acid cycle, Calvin cycle, reductive tricarboxylic acid cycle (rTCA) cycle, 3-hydroxypropionate cycle, reductive acetyl CoA pathway, dicarboxylate-hydroxybutyrate cycle and hydroxypropionate-hydroxybutyrate pathway (Berg et al., 2010). Overall, the carbon fixation genes of C4 dicarboxylic acid, Calvin, reductive TCA cycles were relatively highly utilized in the soils (Figure 7). Proteobacteria contributed significantly to the transcription of genes of the rTCA cycle and Calvin cycle; Nitrospinae also contributed to *porA, porB, porD*, and *porG* (Figure 7, Table S13). Bacteroidetes, Chloroflexi, and Planctomycetes contributed a large proportion to the *ppc* gene expression, and Bacteroidetes, Tectomicrobia, Chloroflexi, Nitrospirae, and Proteobacteria contributed to *idh1* gene transcription (Figure 7, Table S13). Chloroflexi contributed a large proportion to *korA* and *korB* gene expression; Chloroflexi and Bacteroidetes contributed mostly to *korD* and *korG* gene transcription (Figure 7, Table S13). Nitrospinae, Rhodothermaeota, and Firmicutes contributed mostly to the 3-hydroxypropionate cycle including the *accA, accB, accC*, and *accD* genes. The reductive acetyl CoA pathway was less represented based on transcriptome data, and Firmicutes and Proteobacteria contributed to its expression (Table S13). In summary, C4 dicarboxylic acid, Calvin, reductive TCA, and bacteria play crucial roles in the carbon fixation process according to the metatranscriptome data.

## Discussion

### Soil salt quantity shapes the microbial community of tarim saline soils

In these arid and semi-arid regions, soil salinization is a serious worldwide environmental problem (Wang et al., 2008). The Tarim Basin is the largest inland Basin located in northwest China, and experiences low annual precipitation (Zu et al., 2003). Most of the Basin is covered by the Taklimakan desert and oases are formed from rivers originating from the surrounding Mountains (Zu et al., 2003). Under these conditions, soluble salt from the ground floor rises to the surface soils along soil capillaries, causing surface soil salinization (Fan et al., 2011), which is consistent with our data that half of the tested soils reached the salinity standard (Hardie and Doyle, 2012).

Proteobacteria, Firmicutes, Actinobacteria, and Euryarchaeota dominated the soils according to our metagenomic data (Figure 2A); however, predominant species on the metagenomic level were not found to be actively transcribed in the soils (Figure 4A). Furthermore, analysis of the sample correlation according to the detected species and the actively transcribed species showed significant differences according to the metagenomic and metatranscriptomic data among the tested soil samples in Tarim Basin (Figure 5). There are at least three possibilities: (1) only few of these detected species which adapted to the special environment were actively transcribed in Tarim soils, (2) relic DNA in the soils affects the observed richness and composition of microbial communities, and (3) samples were only collected at a single point at each site but there could be temporal changes in the active community that we would not have detected. To explain the first hypothesis, we compared the most active species according to metatranscriptomic data. Although the 10 most abundant based on metagenomic data represented a small proportion of total species (Figure 4A), the 10 most actively transcribed species according to metatranscriptomic data represented a large proportion of total species (Figure 4B). Within the most active species, we found that halophilic microorganisms represented a large proportion of total species (Figure 4C). Our data revealed that actively transcribing halophilic species is consistent with the soil salinities in the tested Tarim soil samples. Previously, it was found environmental factors will shape activities of microbial communities in arid soil systems. For example, in Mediterranean semi-arid soils with rich nutrients, high concentration of dissolved organic carbon shapes the activities of bacterial and fungal populations (Bastida et al., 2016). Halophilic microorganisms are found to be predominant in hot and hypersaline environments such as salted lakes, hot springs, or salted ponds (Bonete et al., 2008; Williams et al., 2014). However, halophilic microorganisms in desert arid conditions have not been well characterized, except for those in the Atacama Desert, Chile (Bull et al., 2016). It is now accepted that soil microbial communities are strongly influenced by environmental factors (Kuramae et al., 2012; Reith et al., 2012). Considering the geochemical data shown in Table 1, we concluded that high soil salinity shaped the microbial communities of Tarim soils such that halophiles are the most actively transcribed species.

However, we cannot exclude relic DNA in the soils affects the observed richness and composition of microbial communities. Recently, it was found that relic DNA remaining in soil after cell death can obscure spatiotemporal patterns and relationships between microbial taxa and environmental conditions, especially in high-pH soils (Carini et al., 2016). The pH values of most tested Tarim soils were relative high (Table 1), which were similar to Mojave desert in southwestern USA (Jordan et al., 1999) and Gobi in southern Mongolia (Kurapova et al., 2012), but higher than many other soils in well-studied desert ecosystems (Makhalanyane et al., 2015). The high pH values of the Tarim soils increases the possibility for remaining relic DNAs in these soils. We cannot also exclude the third possibility that active microbial communities may change over time at a single sampling site. To fully address this possibility and reduce the variability of the microbial diversity at each site, more soil samples should be collected at one site for metagenomic and metatranscriptomic analyses in the further study. Whatever, high salinity and high pH of the tested Tarim soils most possibly shaped the microbial communities and affected their transcriptional activities.

### Archaea are important players in nitrogen cycle in tarim soils

Nitrogen is often regarded as the main factor that limits the productivity of arid terrestrial ecosystems (Hooper and Johnson, 1999). Moreover, both nitrogen and carbon enter desert ecosystems mostly via biological nitrogen and carbon fixation (Belnap, 2002; Belnap and Eldridge, 2003). Previously, it was found archaea seem to be relative abundant in desert soils, with Thaumarcheota being the principle representative (Fierer et al., 2012). However, their ecological functions in nitrogen and carbon metabolisms in arid system is not well studied. In the present study, genes involved in nitrogen cycle and carbon fixation were found to be transcribed in the tested soils. Indeed, archaea, including Thaumarchaeota, Euryarchaeota, and Crenarchaeota, were found to play important roles in these pathways.

Nitrogen cycle includes nitrogen fixation and assimilation, and nitrogen loss in the soils. However, genes of nitrogen fixation were not actively transcribed (Figure 6), suggesting that the biological nitrogen fixation process is impaired in Tarim soils. Although Bacteria, mainly including Proteobacteria and Bacteroidetes, played the most important role in nitrogen cycle, Archaea were found to be the important players. Genes involved in ammonia oxidation of nitrification pathway was weakly transcribed in tested soil samples (Figure 6). However, the *amoABC* genes encoded by Thaumarchaeota were more actively transcribed than the *hao* gene only encoded by bacterial ammonia oxidizers (Kozlowski et al., 2016), suggesting Thaumarchaeota contributed most to ammonia oxidation (Figure 6). Furthermore, we don't identify gene transcripts of hydroxypropionate-hydroxybutyrate cycle from Thaumarchaeota, suggesting ammonia oxidizing Thaumarchaeota might be heterotrophic in Tarim soils.

In assimilatory nitrate reduction pathway, *Halobacteria* of Euryarchaeota contributed most to *nirA* gene expression (Figure 6). In dissimilatory nitrate reduction pathway, Thaumarchaetoa contributed a large proportion of *nirD* and *nrfA* gene expression (Figure 6). Loss of nitrogen is associated with transformation of nitrogen into N_2_, NO, N_2_O or NH_3_ in the denitrification pathway. In this pathway, Thaumarchaeota contributed most to *nirK* gene expression, whereas Crenarchaeota and Euryarchaeota contributed most to *nirK* and *norB* gene expression in some soil samples (Figure 6).

In summary, our study describes the analysis of the microbial community structures of the soils from Tarim Basin, China, based on metagenomic and metatranscriptomic data. We identified the most abundant and most active phylum-level OTUs and found halophiles were highly represented based on their abundant transcripts, suggesting the active microbial communities are consistent with the geochemistry of the Tarim soils. Our transcriptomic data also indicated the microbial genes involved in nitrogen cycling and carbon fixation were expressed at variable levels, suggesting diverse microbial communities could provide carbon and nitrogen nutrients for higher plants in the sandy saline soils of Tarim Basin.

## Author contributions

MR: sampled the soils and analyzed the data; ZhuZ and CZ: analyzed the data and wrote the manuscript; XW, DC, SZ, and HZ: extracted and sequenced DNA and RNA, and analyzed the data; ZhiZ, LC, CZ, and YZ: analyzed the data wrote the manuscript; YL, QS, and NP: designed the project, analyzed the data and wrote the manuscript. All authors approve to publish this paper and agree to be accountable for all aspects of the work in ensuring that questions related to the accuracy or integrity of any part of the work.

### Conflict of interest statement

The authors declare that the research was conducted in the absence of any commercial or financial relationships that could be construed as a potential conflict of interest.
