# Supplementary material for: Diversity and Contributions to Nitrogen Cycling and Carbon Fixation of Soil Salinity Shaped Microbial Communities in Tarim Basin
Source: Front Microbiol. 2018 Mar 9;9:431. doi: 10.3389/fmicb.2018.00431 (PMC5855357; doi:10.3389/fmicb.2018.00431)
Supplement: Supplementary file 1 [file DataSheet1.ZIP › 317810_Min _Data_Sheet_1_0223/Supplementary data-rm/Table S1. Details of the sample locations..docx]

Table S1. Details of the sample locations.

| **Sample** | **Location** | **Latitude (N)** | **Longitude (E)** | **Altitude (m)** |
| --- | --- | --- | --- | --- |
| A1 | Hotan | 37°51′26.124″ | 80°29′32.518″ | 1186.17 |
| A2 |  | 37°51′32.346″ | 80°29′35.271″ |  |
| A3 |  | 37°51′24.271″ | 80°29′32.96″ |  |
| B1 | Hotan | 37°27′54.619″ | 79°34′20.211″ | 1292 |
| B2 |  | 37°27′58.45″ | 79°34′25.814″ |  |
| B3 |  | 37°27′45.637″ | 79°34′16.833″ |  |
| C1 | Hotan | 37°34′58.512″ | 80°08′48.662″ | - |
| C2 |  | 37°34′58.499″ | 80°08′51.271″ |  |
| C3 |  | 37°35′1.108″ | 80°08′50.145″ |  |
| D1 | Xayar | 41°02′15.077″ | 82°32′59.242″ | 962 |
| D2 |  | 41°02′14.129″ | 82°32′54.380″ | 963 |
| D3 |  | 41°02′15.146″ | 82°32′54.023″ | 966 |
| E1 | Xayar | 41°09′35.067″ | 82°56′29.240″ | 954 |
| E2 |  | 41°09′36.021″ | 82°56′26.129″ |  |
| E3 |  | 41°09′36.821″ | 82°56′33.612″ |  |
| F1 | Xayar | 40°55′10.153″ | 83°07′46.672″ | 950 |
| F2 |  | 40°55′14.575″ | 83°07′44.529″ | 952 |
| F3 |  | 40°55′14.919″ | 83°07′41.838″ | 953 |

| **Sample** | **Location** | **Latitude (N)** | **Longitude (E)** | **Altitude (m)** |
| --- | --- | --- | --- | --- |
| A1 | Hotan | 37°51′26.124″ | 80°29′32.518″ | 1186.17 |
| A2 |  | 37°51′32.346″ | 80°29′35.271″ |  |
| A3 |  | 37°51′24.271″ | 80°29′32.96″ |  |
| B1 | Hotan | 37°27′54.619″ | 79°34′20.211″ | 1292 |
| B2 |  | 37°27′58.45″ | 79°34′25.814″ |  |
| B3 |  | 37°27′45.637″ | 79°34′16.833″ |  |
| C1 | Hotan | 37°34′58.512″ | 80°08′48.662″ | - |
| C2 |  | 37°34′58.499″ | 80°08′51.271″ |  |
| C3 |  | 37°35′1.108″ | 80°08′50.145″ |  |
| D1 | Xayar | 41°02′15.077″ | 82°32′59.242″ | 962 |
| D2 |  | 41°02′14.129″ | 82°32′54.380″ | 963 |
| D3 |  | 41°02′15.146″ | 82°32′54.023″ | 966 |
| E1 | Xayar | 41°09′35.067″ | 82°56′29.240″ | 954 |
| E2 |  | 41°09′36.021″ | 82°56′26.129″ |  |
| E3 |  | 41°09′36.821″ | 82°56′33.612″ |  |
| F1 | Xayar | 40°55′10.153″ | 83°07′46.672″ | 950 |
| F2 |  | 40°55′14.575″ | 83°07′44.529″ | 952 |
| F3 |  | 40°55′14.919″ | 83°07′41.838″ | 953 |
