# Supplementary material for: Diversity and Contributions to Nitrogen Cycling and Carbon Fixation of Soil Salinity Shaped Microbial Communities in Tarim Basin
Source: Front Microbiol. 2018 Mar 9;9:431. doi: 10.3389/fmicb.2018.00431 (PMC5855357; doi:10.3389/fmicb.2018.00431)
Supplement: Supplementary file 1 [file DataSheet1.ZIP › 317810_Min _Data_Sheet_1_0223/Supplementary data-rm/Table S2 ANOVA result of chemistry characteristics and diversity.docx]

Table S2: The ANOVA results of chemistry characteristics and diversity. The values in the table are the p-values by two-way ANOVA method. Significant difference was highlighted by bold font.

| Subjects | Location factor | Site factor | Interaction |
| --- | --- | --- | --- |
| Conductivity | 0.2790 | 0.6928 | 0.9523 |
| Dissolved organic carbon | **0.0025** | 0.4005 | 0.2737 |
| Total carbon | **0.0003** | 0.0976 | **0.0492** |
| pH | 0.4544 | 0.9192 | 0.8518 |
| Total nitrogen | **0.0175** | 0.8490 | 0.9223 |
| Nitrate nitrogen | **0.0063** | **0.0226** | 0.7352 |
| Ammonium nitrogen | 0.0832 | 0.1419 | 0.0964 |
| Exchangeable potassium | 0.3313 | 0.9429 | 0.6289 |
| Exchangeable sodium | 0.1685 | 0.2206 | 0.4972 |
| Exchangeable calcium | **0.0008** | 0.6691 | 0.7169 |
| Exchangeable magnesium | 0.1312 | 0.8084 | 0.8951 |
|  |  |  |  |
| Alpha diversity | 0.1342 | 0.6585 | 0.8255 |
| Beta diversity | 0.0529 | 0.3266 | 0.7167 |
|  |  |  |  |
