# Supplementary material for: Diversity and Contributions to Nitrogen Cycling and Carbon Fixation of Soil Salinity Shaped Microbial Communities in Tarim Basin
Source: Front Microbiol. 2018 Mar 9;9:431. doi: 10.3389/fmicb.2018.00431 (PMC5855357; doi:10.3389/fmicb.2018.00431)
Supplement: Supplementary file 1 [file DataSheet1.ZIP › 317810_Min _Data_Sheet_1_0223/Supplementary data-rm/Table S3. Statistical analysis of assembled contigs from metagenomic DNA..docx]

Table S3. Statistical analysis of assembled contigs from metagenomic DNA.

| Sample | Raw data | Clean reads | CleanPer | Unique reads | Contig | MaxLen | N50 | Mean | Total base | Used reads |
| --- | --- | --- | --- | --- | --- | --- | --- | --- | --- | --- |
|  |  |  |  |  |  |  |  |  |  |  |
| A1 | 41168556 | 35544130 | 86.34% | 35129229(98.83%) | 10169173 | 3900 | 133 | 107 | 1088987640 | 25737528/35544130 |
| A2 | 45659960 | 38696243 | 84.75% | 38278194(98.92%) | 11192767 | 4766 | 130 | 100 | 1124268336 | 27449725/38696243 |
| A3 | 39168124 | 33849997 | 86.42% | 33492086(98.94%) | 9916016 | 4039 | 131 | 111 | 1102212300 | 23491080/33849997 |
| B1 | 39135274 | 33770825 | 86.29% | 33407796(98.93%) | 9559800 | 3023 | 135 | 113 | 1085520920 | 23724440/33770825 |
| B2 | 45655082 | 38709642 | 84.79% | 38341721(99.05%) | 10804515 | 4305 | 135 | 106 | 1148089196 | 27017988/38709642 |
| B3 | 46348792 | 39411018 | 85.03% | 38996728(98.95%) | 10117046 | 5597 | 137 | 113 | 1147456953 | 28417482/39411018 |
| C1 | 43459238 | 37199273 | 85.60% | 36811415(98.96%) | 10956303 | 4408 | 131 | 110 | 1210398835 | 25476654/37199273 |
| C2 | 44466932 | 37852292 | 85.12% | 37461734(98.97%) | 10929671 | 5535 | 133 | 113 | 1244336432 | 24490304/37852292 |
| C3 | 44631020 | 37915833 | 84.95% | 37501749(98.91%) | 10999096 | 5295 | 132 | 106 | 1175453429 | 26318625/37915833 |
| D1 | 45741824 | 38580518 | 84.34% | 38264583(99.18%) | 11230012 | 2885 | 134 | 107 | 1212201528 | 24622747/38580518 |
| D2 | 43197424 | 37626825 | 87.10% | 37081570(98.55%) | 12373936 | 5004 | 129 | 98 | 1214455812 | 25213675/37626825 |
| D3 | 44605692 | 38422815 | 86.14% | 38000306(98.90%) | 10819403 | 6169 | 137 | 108 | 1171516222 | 27232005/38422815 |
| E1 | 40731246 | 35201199 | 86.42% | 33426256(94.96%) | 16368652 | 3376 | 133 | 58 | 957661583 | 20975167/35201199 |
| E2 | 43241594 | 36775644 | 85.05% | 36350894(98.85%) | 10306517 | 5911 | 131 | 107 | 1105286421 | 26201929/36775644 |
| E3 | 43815322 | 37218289 | 84.94% | 36771134(98.80%) | 10500911 | 5608 | 129 | 104 | 1093159242 | 26851717/37218289 |
| F1 | 46725978 | 39478261 | 84.49% | 39028962(98.86%) | 11762403 | 2253 | 131 | 102 | 1206086041 | 26384652/39478261 |
| F2 | 39823270 | 33928173 | 85.20% | 33566765(98.93%) | 9738818 | 4087 | 129 | 104 | 1018933317 | 24174194/33928173 |
| F3 | 45937212 | 38797112 | 84.46% | 38409495(99.00%) | 11343519 | 3632 | 133 | 110 | 1257952768 | 25236433/38797112 |
