# Supplementary material for: Diversity and Contributions to Nitrogen Cycling and Carbon Fixation of Soil Salinity Shaped Microbial Communities in Tarim Basin
Source: Front Microbiol. 2018 Mar 9;9:431. doi: 10.3389/fmicb.2018.00431 (PMC5855357; doi:10.3389/fmicb.2018.00431)
Supplement: Supplementary file 1 [file DataSheet1.ZIP › 317810_Min _Data_Sheet_1_0223/Supplementary data-rm/Table S4. Statistical analysis of assembled contigs from metatranscriptomic RNA..docx]

Table S4. Statistical analysis of assembled contigs from metatranscriptomic RNA.

| Sample | Raw data | Clean reads | CleanPer | Uniqtag | Contig | MaxLen | N50 | Mean |
| --- | --- | --- | --- | --- | --- | --- | --- | --- |
|  |  |  |  |  |  |  |  |  |
| A1 | 18356200 | 10120298 | 55.13% | 3668086(36.24%) | 48285 | 6116 | 237 | 239 |
| A2 | 34453752 | 31533039 | 91.52% | 21906036(69.47%) | -- | -- | -- | -- |
| A3 | 17906774 | 14074872 | 78.60% | 4504114(32.00%) | 35998 | 4865 | 234 | 236 |
| B1 | 19211292 | 17377655 | 90.46% | 6020855(34.65%) | 169774 | 5832 | 235 | 240 |
| B2 | 22191668 | 20150920 | 90.80% | 11717793(58.15%) | 496564 | 10400 | 246 | 263 |
| B3 | 30027354 | 26009310 | 86.62% | 17211300(66.17%) | -- | -- | -- | -- |
| C1 | 37706932 | 35080747 | 93.04% | 21034386(59.96%) | 412852 | 16465 | 270 | 289 |
| C2 | 16523372 | 15136600 | 91.61% | 9434598(62.33%) | 291650 | 9895 | 240 | 250 |
| C3 | 16479840 | 14862568 | 90.19% | 9277717(62.42%) | 490650 | 32633 | 245 | 260 |
| D1 | 13698064 | 12301555 | 89.81% | 7879812(64.06%) | 350350 | 6115 | 243 | 251 |
| D2 | 16537696 | 14260311 | 86.23% | 9472076(66.42%) | 483633 | 6064 | 242 | 254 |
| D3 | 30961606 | 28911833 | 93.38% | 20823601(72.02%) | 562138 | 32111 | 292 | 309 |
| E1 | 18143902 | 15938598 | 87.85% | 9305760(58.39%) | -- | -- | -- | -- |
| E2 | 28551304 | 26179217 | 91.69% | 18485089(70.61%) | -- | -- | -- | -- |
| E3 | 20738576 | 15839336 | 76.38% | 9337421(58.95%) | 324532 | 6431 | 246 | 257 |
| F1 | 19026424 | 16184337 | 85.06% | 10657760(65.85%) | 350461 | 7825 | 251 | 268 |
| F2 | 20181802 | 18440721 | 91.37% | 14171354(76.86%) | 517095 | 51810 | 299 | 317 |
| F3 | 19312796 | 16466659 | 85.26% | 9404098(57.11%) | -- | -- | -- | -- |
