# Supplementary material for: Diversity and Contributions to Nitrogen Cycling and Carbon Fixation of Soil Salinity Shaped Microbial Communities in Tarim Basin
Source: Front Microbiol. 2018 Mar 9;9:431. doi: 10.3389/fmicb.2018.00431 (PMC5855357; doi:10.3389/fmicb.2018.00431)
Supplement: Supplementary file 1 [file DataSheet1.ZIP › 317810_Min _Data_Sheet_1_0223/Supplementary data-rm/Table S5. Distribution of different Domins by MetaPhlAn analysis based on metagenomic sequences..docx]

Table S5. Distribution of different Domains by MetaPhlAn analysis based on metagenomic sequence.

| **Sample** | **Archaea** | **Bacteria** | **Eukaryota** | **Viroids** |
| --- | --- | --- | --- | --- |
| A1 | 7.37% | 90.12% | 2.25% | 0.26% |
| A2 | 9.07% | 88.17% | 2.13% | 0.62% |
| A3 | 7.75% | 88.53% | 2.23% | 1.50% |
| B1 | 8.79% | 88.51% | 2.09% | 0.61% |
| B2 | 8.57% | 88.66% | 1.99% | 0.77% |
| B3 | 6.64% | 90.77% | 2.01% | 0.58% |
| C1 | 6.84% | 90.26% | 2.13% | 0.76% |
| C2 | 6.71% | 90.05% | 2.29% | 0.96% |
| C3 | 10.94% | 86.46% | 1.94% | 0.66% |
| D1 | 6.54% | 91.14% | 1.81% | 0.51% |
| D2 | 6.69% | 90.07% | 2.39% | 0.84% |
| D3 | 6.95% | 90.28% | 2.26% | 0.51% |
| E1 | 6.28% | 89.48% | 3.24% | 1.00% |
| E2 | 6.83% | 90.11% | 2.13% | 0.93% |
| E3 | 6.63% | 90.26% | 2.41% | 0.71% |
| F1 | 6.62% | 90.42% | 2.23% | 0.73% |
| F2 | 6.71% | 90.85% | 2.07% | 0.37% |
| F3 | 6.52% | 90.83% | 2.11% | 0.53% |
| Average | 7.36% | 89.72% | 2.21% | 0.71% |
