# Supplementary material for: Diversity and Contributions to Nitrogen Cycling and Carbon Fixation of Soil Salinity Shaped Microbial Communities in Tarim Basin
Source: Front Microbiol. 2018 Mar 9;9:431. doi: 10.3389/fmicb.2018.00431 (PMC5855357; doi:10.3389/fmicb.2018.00431)
Supplement: Supplementary file 1 [file DataSheet1.ZIP › 317810_Min _Data_Sheet_1_0223/Supplementary data-rm/Table S7. Distribution of different Domains by MetaPhlAn analysis accroding to metatranscriptomic sequences..docx]

Table S7 Distribution of different Domains by MetaPhlAn analysis according to metatranscriptomic sequences

| **Sample** | **Archaea** | **Bacteria** | **Eukaryota** | **Viroids** |
| --- | --- | --- | --- | --- |
| A1 | 0.57% | 96.70% | 2.74% | 0.00% |
| A2 | 25.25% | 73.26% | 1.49% | 0.00% |
| A3 | 0.50% | 99.46% | 0.05% | 0.00% |
| B1 | 15.62% | 6.55% | 0.00% | 77.83% |
| B2 | 4.18% | 95.79% | 0.03% | 0.00% |
| B3 | 7.44% | 88.84% | 3.71% | 0.00% |
| C1 | 6.04% | 90.12% | 1.32% | 2.52% |
| C2 | 12.29% | 87.69% | 0.02% | 0.00% |
| C3 | 39.07% | 60.93% | 0.00% | 0.00% |
| D1 | 0.63% | 99.37% | 0.00% | 0.00% |
| D2 | 0.74% | 99.25% | 0.01% | 0.00% |
| D3 | 14.27% | 79.42% | 3.87% | 2.45% |
| E1 | 0.05% | 99.95% | 0.00% | 0.00% |
| E2 | 9.13% | 88.05% | 0.74% | 2.08% |
| E3 | 6.31% | 86.63% | 0.03% | 7.02% |
| F1 | 2.13% | 97.57% | 0.30% | 0.00% |
| F2 | 2.56% | 95.88% | 0.00% | 1.55% |
| F3 | 0.71% | 99.28% | 0.01% | 0.00% |
| Average | 7.76% | 90.48% | 0.84% | 0.92% |
